# Correction: Experimental investigation of Mg(B_3_H_8_)_2_ dimensionality, materials for energy storage applications

**DOI:** 10.1039/d2dt90095e

**Published:** 2022-06-08

**Authors:** Romain Moury, Angelina Gigante, Arndt Remhof, Elsa Roedern, Hans Hagemann

**Affiliations:** Institut des Molécules et des Matériaux du Mans, UMR 6283 CNRS, Le Mans Université Avenue Olivier Messiaen 72085 Le Mans Cedex 9 France; Dépt. de Chimie Physique, Université de Genève 30 quai E. Ansermet CH 1211 Geneva 4 Switzerland; Empa, Swiss Federal Laboratories for Materials Science and Technology Überlandstrasse 129 8600 Dübendorf Switzerland

## Abstract

Correction for ‘Experimental investigation of Mg(B_3_H_8_)_2_ dimensionality, materials for energy storage applications’ by Romain Moury *et al.*, *Dalton Trans.*, 2020, **49**, 12168–12173, https://doi.org/10.1039/D0DT02170A.

The authors would like to inform the readers that ref. 1 below is missing in the original article. Ref. 1 deals with the synthesis of Mg(B_3_H_8_)_2_ using a similar procedure.

The Royal Society of Chemistry apologises for these errors and any consequent inconvenience to authors and readers.

## Supplementary Material

## References

[cit1] Kim D. Y., Yang Y., Abelson J. R., Girolami G. S. (2007). Inorg. Chem..

